# The Use of Electronic Consultations in Outpatient Surgery Clinics: Synthesized Narrative Review

**DOI:** 10.2196/34661

**Published:** 2022-04-14

**Authors:** Thomas Payne, Jasmina Kevric, Wanda Stelmach, Henry To

**Affiliations:** 1 Faculty of Medicine, Dentistry and Health Sciences The University of Melbourne Melbourne Australia; 2 Department of Surgery The Northern Hospital Melbourne Australia; 3 Department of Surgery Werribee Mercy Hospital Melbourne Australia

**Keywords:** telemedicine, telehealth, electronic consultation, electronic referral, surgery, outpatient, consultation, usage, review, referral, advice, communication, framework, efficacy, safety, limit

## Abstract

**Background:**

Electronic consultations (eConsults) are an increasingly used form of telemedicine that allows a nonspecialist clinician to seek specialist advice remotely without direct patient-specialist communication. Surgical clinics may see benefits from such forms of communication but face challenges with the need for intervention planning.

**Objective:**

We aimed to use the Quadruple Aim Framework to integrate published knowledge of surgical outpatient eConsults with regard to efficacy, safety, limitations, and evolving use in the era of COVID-19.

**Methods:**

We systematically searched for relevant studies across four databases (Ovid MEDLINE, Embase, Scopus, and Web of Science) on November 4, 2021, with the following inclusion criteria: English language, published in the past 10 years, and data on the outcomes of outpatient surgical eConsults.

**Results:**

A total of 363 studies were screened for eligibility, of which 33 (9.1%) were included. Most of the included studies were from the United States (23/33, 70%) and Canada (7/33, 21%), with a predominant multidisciplinary focus (9/33, 27%). Most were retrospective audits (16/33, 48%), with 15% (5/33) of the studies having a prospective component.

**Conclusions:**

The surgical eConsult studies indicated a possible benefit for population health, promising safety results, enhanced patient and clinician experience, and cost savings compared with the traditional face-to-face surgical referral pathway. Their use appeared to be more favorable in some surgical subspecialties, and the overall efficacy was similar to that of medical subspecialties. Limited data on their long-term safety and use during the COVID-19 pandemic were identified, and this should be the focus of future research.

## Introduction

### Background

The SARS-CoV-2 pandemic has potentiated an increased uptake of telemedicine by health practitioners [1-4]. Telemedicine refers to a broad range of electronic services that obviate the need for face-to-face interactions but maintain the same patient-physician relationship [5-10].

An emerging component of telemedicine is electronic consultations (eConsults). eConsults are asynchronous clinician-to-clinician consultations via a secure web-based platform. They allow a primary care provider (PCP), such as a physician, nurse practitioner, or physician assistant, to seek nonurgent specialist advice remotely without direct contact between the patient and specialist [11]. eConsults may be used to replace an in-person consultation or ensure that an appropriate workup is completed before a face-to-face visit. They represent a well-documented, asynchronous replacement of the *curbside* consultation. eConsults differ from electronic triage systems that prioritize the urgency of patient-specialist consults rather than replacing them.

**Figure 1:**
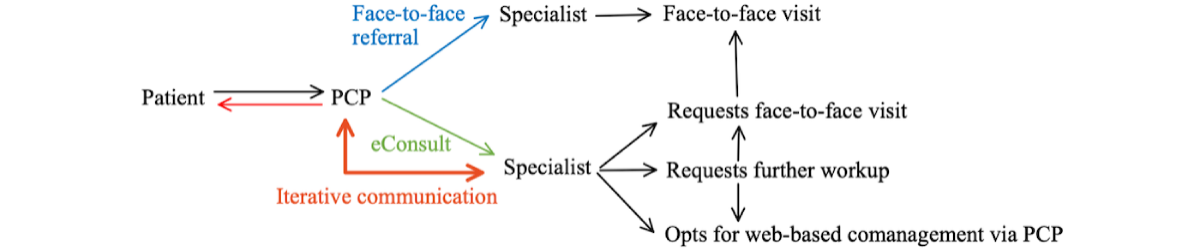
Basic flowchart of the *optional-pathway* electronic consultation (eConsult) referral process. Primary care providers (PCP) are given a choice between referring via a traditional in-person visit or via an eConsult.

**Figure 2:**
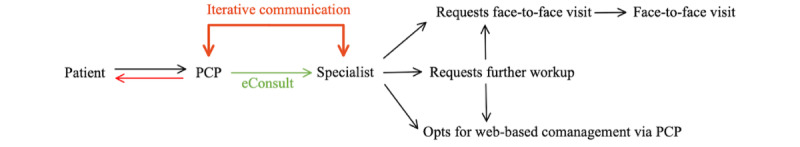
Basic flowchart of the *single-pathway* electronic consultation (eConsult) referral process. In this structure, all specialist referrals are submitted as eConsults. PCP: primary care provider.

The entry point for the PCP into the eConsult system depends on the structure of the health service [12]. In *optional-pathway* services, the PCP is able to choose to refer their patient via a face-to-face pathway or via an eConsult. Conversely, in *single-pathway* services, the PCP must refer the patient via an eConsult, and the specialist can then decide if a subsequent face-to-face visit is necessary (Figures 1 and 2) [13-16]. Some eConsult services set clear criteria, such as mandatory investigations before lodging an eConsult. The information returned from the specialist via the eConsults provides the PCP with assistance with diagnosis, imaging interpretation, and advice on management [17]. The web-based communication platform may be a health service–specific program, or a shared electronic medical record (EMR), where the correspondence is recorded [18,19]. Outcomes include scheduling a face-to-face appointment if required or giving management advice virtually. In some cases, eConsults undergo an iterative process in which the specialist requests further information before making a decision.

eConsults address the limitations of the current medical system. From an equity perspective, disadvantaged demographics [20-23] can engage specialists, as eConsults are more economical for these groups, by bypassing social barriers and reducing travel and work expenses [24,25]. eConsults can address lengthy specialist wait times [26] to obviate specific bottlenecks in the referral pathway [27]. The lack of physical contact in eConsults means that they can still operate even within social distancing restrictions [28-31].

The feasibility of eConsults in medicine has been studied extensively [32-38]; however, their role in the surgical stream is less well-understood [39-41]. Surgical and medical eConsults differ in the variety of conditions, and those surgical conditions may require an intervention. At face value, this may imply that surgical subspecialties lend themselves less to eConsults as a face-to-face visit may be inevitable for assessment and consent [42]. Multispecialty studies have shown different patterns of eConsult use in medical and surgical conditions. For example, the eConsult requester seemed more likely to be a nurse practitioner familiar with surgical compared with medical eConsults [43]. Another study suggested that PCPs deem surgical eConsults to be of lower quality than medical eConsults [44].

### Objectives

The aim of this review is to build on previous systematic reviews of eConsults by focusing specifically on utility and outcomes in surgical outpatients. We synthesize our assessment using the Quadruple Aim Framework [45], which helps guide the assessment of ideal health service performance outcomes. The four components of this framework are (1) improving the health of the population, (2) enhancing patient experience of care, (3) reducing per capita cost of health care, and (4) improving the work life of health care clinicians and staff. These 4 goals are interrelated and serve to maximize the primary goal of improving population health. The role of the Quadruple Aim Framework in eConsult evaluation has been established elsewhere [12,46] and was used in a recent eConsult systematic review [40]. For the purposes of this review, we use *clinicians* to refer to PCPs and specialists.

## Methods

### Protocol

This study used a narrative review with a systematic approach. We used the PRISMA (Preferred Reporting Items for Systematic Reviews and Meta-Analyses) guidelines to conduct the search strategy.

### Search Strategy

On November 4, 2021, we conducted a search of four databases (Ovid MEDLINE, Ovid Embase, Web of Science, and Scopus). The reference lists of review articles were scanned for additional studies. Electronic referrals (*eReferrals*) differ slightly from *eConsults* in that their primary goal is to expedite a patient’s workup before an in-person specialist visit; however, terminology in this field is variable [47], and, for the purpose of this review, these terms were combined. Thus, the search terms were one for eConsults (eg, *eConsult* and *eReferral*) and one for surgical subspecialties (eg, *surgery* and *orthopaedics*; Multimedia Appendix 1). The search was limited to articles in English and those published in the past 10 years (2011-2021), given that most modern eConsult platforms were studied after 2010.

### Eligibility Criteria

The inclusion criteria were studies on outpatient surgical eConsults, eReferrals, and store-and-forward telemedicine consults that included dedicated surgical articles and articles that included surgical eConsults as a subanalysis of a multispecialty cohort. We only included eReferral services that allowed for iterative PCP-specialist communication. We only included original studies (including observational and experimental studies) of outpatients. We excluded studies on asynchronous clinician-to-clinician communication that did not use an appropriate platform for shared patient information (eg, surgical wound images). We excluded all conference abstracts, case reports, editorials, notes, and letters.

### Study Selection

The titles and abstracts of the obtained articles were screened by an investigator (TP) as per the inclusion criteria. Moreover, 2 investigators (TP and HT) critically appraised all the included articles independently. Disagreements regarding article inclusion were resolved through discussion between the 2 investigators.

### Data Extraction

Data were extracted into a Microsoft Excel spreadsheet independently by 2 investigators (TP and HT) into categories corresponding to the relevant study objectives. Extracted data included study variables (author, title, year, country, and surgical subspecialty), eConsult service design, study outcome data, and study conclusions.

### Narrative Synthesis

A narrative synthesis was more appropriate than other synthesis methods (including meta-analysis), given the significant heterogeneity in eConsult service designs and outcome measures and the overlapping eConsult data between studies [48]. The data were synthesized by grouping together similar outcome metrics across all studies to provide a range and by grouping surgical subspecialties together to compare findings across and within different fields. The obtained narrative synthesis information was subdivided into sections using the Quadruple Aim Framework as a guide. In cases where systematic reviews were identified, we extracted key discussion points to include in our study. The findings of our narrative synthesis were depicted in tabular and schematic diagram form. Individual appraisals of study quality were not performed because of time and personnel constraints and were beyond the scope of this review.

## Results

### Overview

**Figure 3:**
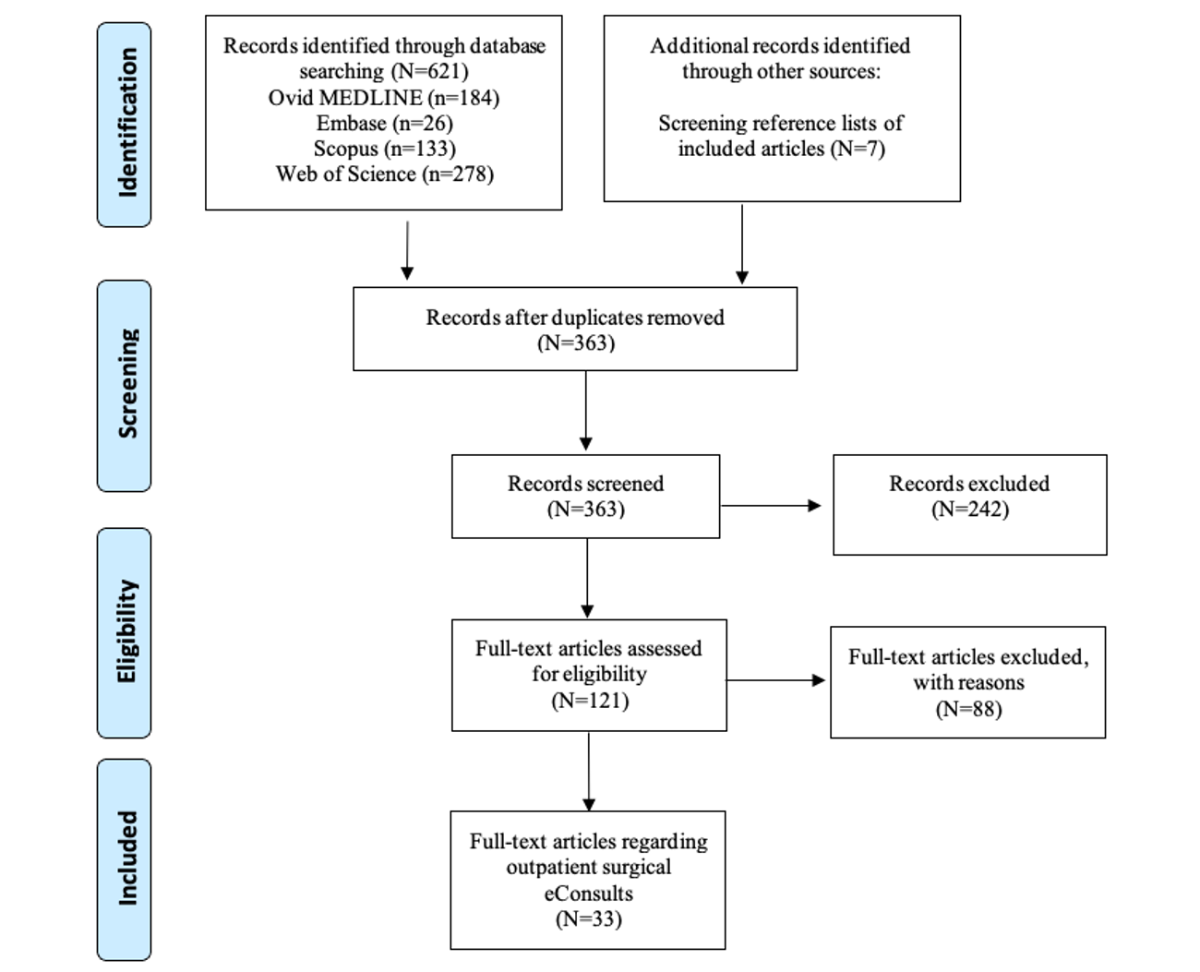
PRISMA (Preferred Reporting Items for Systematic Reviews and Meta-Analyses) flowchart of the included studies. eConsult: electronic consultation.

A total of 33 studies were included (Figure 3). The characteristics of the studies analyzed are outlined in Table 1, and detailed descriptions of the studies are provided in Multimedia Appendix 2 [11,18,19,27,39,40,43,44,49-73]. Most of the included studies were published in the past 5 years (26/33, 79%), and half were from surgical journals (17/33, 52%), mainly from North America (30/33, 91%). eConsults were most represented in urology, otolaryngology, and obstetrics and gynecology.

Most of the included studies used a retrospective audit of EMR data with or without a mandatory PCP exit survey. Most of these studies used data from a single health service network, and only 1 of the studies combined surgical eConsult data from multiple hospital platforms [49]. Of the retrospective studies, 12% (4/33) were pre- versus postimplementation studies [18,27,50,51]. Approximately 3% (1/33) of the studies used feedback from post-eConsult surveys to assess the ability of the specialist to incorporate this feedback into their practice [44]. Only 15% (5/33) of the studies used a prospective design in their analysis [27,44,50,52,53]. None of these studies randomized patients to an eConsult or a face-to-face visit. Most studies assessed clinician satisfaction, with only 6% (2/33) of the studies assessing patient satisfaction with surgical eConsults [11,53].

**Table 1:** General characteristics of the included studies (N=33).

Characteristic		Studies, n (%)
**Year of publication**		
	2010-2013	3 (9)
	2014-2015	4 (12)
	2016-2017	7 (21)
	2018-2019	9 (27)
	2020-2021	10 (30)
**Country of origin**		
	United States	23 (70)
	Canada	7 (21)
	New Zealand	1 (3)
	Nigeria	1 (3)
	Spain	1 (3)
**Surgical subspecialty**		
	Urology	7 (21)
	Otolaryngology	3 (9)
	Obstetrics and gynecology	3 (9)
	Orthopedics	2 (6)
	Pediatric surgery	2 (6)
	General surgery	2 (6)
	Vascular surgery	2 (6)
	Anesthesiology	1 (3)
	Maxillofacial	1 (3)
	Neurosurgery	1 (3)
	Multispecialty	9 (27)
**Type of journal**		
	Surgical	17 (52)
	Health services	7 (21)
	Medical informatics	5 (15)
	Medicine	3 (9)
	General	1 (3)
**Type of article**		
	Retrospective audit	16 (48)
	Mixed methods—retrospective audit+survey	7 (21)
	Prospective observational cohort study	4 (12)
	Systematic review	3 (9)
	Mixed methods—retrospective audit+prospective study	1 (3)
	Cross-sectional+qualitative study	1 (3)
	Survey	1 (3)

### Improving Population Health

#### Pattern of eConsult Use Among Clinicians

Surgical eConsult use is increasing [27,43,54-57]—one of the studies showed a 50-fold increase in annual eConsults over their 3-year study period (103 is 2012 vs 5023 in 2015) [55]. In *optional-pathway* services, surgical eConsults constituted a minority of the total surgical referrals, ranging from 1.8% to 5.8% [11,43,58]. Although this suggests that PCPs still prefer face-to-face referrals for surgical conditions, it is likely because of the relatively recent introduction of eConsults; some telehealth initiatives implemented during the SARS-CoV-2 pandemic have received widespread support from clinicians to remain in place [74]. Overall, eConsults were used less frequently for surgical compared with medical conditions [43,50,58-60]. However, Saxon et al [43] found that the percentage of surgical referrals that were eConsults was increasing at almost twice the rate compared with medical referrals, suggesting that PCPs have become more comfortable referring surgical patients, with 6% (2/33) of the studies suggesting that eConsults were replacing face-to-face visits altogether [51,61]. The most frequent subspecialties to use eConsults were orthopedics in 6% (2/33) of the studies [50,59], otolaryngology and obstetrics and gynecology in 3% (1/33) of the studies [60], and preoperative evaluation in another study [43]. Uptake was variable across surgical subspecialties partly because of PCPs’ exposure to the subspecialty—Parikh et al [49] found that 62.7% (69/110) of patients of neurosurgery versus 12.3% (74/600) of patients with diabetes mellitus were referred to specialists as eConsults, suggesting PCPs seek virtual guidance for presentations with which they have less experience in management. Most studies suggested that management inquiries were the most common reason for the eConsult [52,62,63].

For outcomes, 9% (3/33) of the studies showed that PCPs adopted a new course of action in 20% to 62% of surgical eConsults [52,63,64]. In the remaining cases, the eConsult still served to reinforce current management. Liddy et al [60] found that new or additional actions were recommended less in the surgical stream compared with almost all the medical subspecialties. This finding may again be a reflection of PCP inexperience with surgical subspecialties; hence, they seek the reassurance of their management plan more frequently than for the more common medical presentations.

#### Patient Safety With eConsults Compared With the Traditional Referral Pathway

A unique safety concern in surgical eConsults was the use of virtual comanagement in cases where an operation was necessary. Only 12% (4/33) of the studies reported safety end points. Castaneda et al [65] found no difference in 5-year mortality between patients who had an eConsult versus the general population data. Another study on patients with vascular conditions found no cases of death or hospitalization in 54 eConsult patients over a 90-day period [11]. In a 2-year study on 1013 very low-risk patients of gynecology, 14.5% (147/1013) were rereferred for a face-to-face visit within 6 months because of ongoing issues for the same condition; however, none had a malignancy attributable to the presenting complaint, and there were no deaths over the 2 years [61]. A study on general surgery eConsults found that 11% (4/36) of virtually managed patients required emergency department care [66]. In total, 2 of these patients who were hospitalized had both been scheduled for additional diagnostic workup before a face-to-face visit, suggesting that no patients had a worse outcome from virtual comanagement. Promisingly, no study identified an increase in adverse outcomes with surgical eConsults, despite this being raised as a concern in patient and clinician surveys [75].

Approximately 12% (4/33) of the studies looked indirectly at patient safety. In 3% (1/33) of the studies, contingency for rereferral was made in 45.7% (160/350) of eConsults [65], and another study found that 43% (30/69) of neurosurgical eConsults showed no documentation of PCP follow-up [49]. Accordingly, surgical eConsult proformas, including rereferral plans and automated safeguards to ensure follow-up, need to be integrated into health systems. A third study found that specialists who dealt with a higher volume of eConsults spent less time responding per eConsult [44], suggesting that services that allow for manageable loads for each specialist may indirectly increase patient safety by freeing up the time of the specialist. The final study found that eConsults used in preoperative evaluation had no significant effect on preventable operation cancellation rates during their 5-year study period [55]. This implies that eConsults do not risk suboptimal care from surgery cancellations; however, the postoperative outcomes of preoperative eConsult patients were not studied. We could not identify any studies that used eConsults for routine surgical follow-up. A recent study demonstrated that early postoperative PCP follow-up was associated with a 47% decreased risk of hospital readmission at 30 days in high-risk patients with surgical complications, and eConsults can further augment this benefit by facilitating prehospital specialist input [76]. eConsults achieve this by improving PCP-hospital communication, given that rates of PCP-specialist communication are very low after discharge, and issues with communication have been shown to negatively affect the care of up to 25% of recently discharged patients [77].

eConsults can promote safety. The studies on *single-pathway* eConsult services (4/33, 12%) showed that 3.1% to 17% of eConsults deemed by the PCP to not require a face-to-face evaluation were changed to an in-person evaluation [50,52,62,64]. These patients would not have been seen by a specialist in the absence of an eConsult and possibly had better long-term outcomes because of the safety net of an eConsult service.

Despite these reassuring safety findings, as also observed by Vimalananda et al [39], we could not identify any studies that listed adverse events as the primary outcome. Furthermore, there were no long-term safety data, with the longest follow-up period being 5 years, and no studies that included complications treated by other health services. The lack of randomization in all studies and the triage of low-risk patients to eConsults and high-risk patients to in-person consultations mean that safety data are skewed away from patients who are more complex and sicker. Another safety concern not addressed in the data is the outcomes of patients treated solely by the PCP using specialist advice from eConsults of previous patients with a similar surgical condition.

#### Surgical Yield of eConsults

Ulloa et al [66] examined the surgical yield of eConsults, defined as the proportion of face-to-face specialist visits that are subsequently scheduled for surgery. Surgical yield is a reflection of the ability to triage patients requiring nonoperative management before a face-to-face visit, which can be improved by eConsults. Surgical yield is an important efficiency measure for surgical services, which has significant funding implications. Note that high rates of surgical yield are condition specific and may imply that there were patients not seen face to face who required surgery. The authors found that there was a nonsignificant trend in favor of eConsults increasing surgical yield (46% (53/114) vs 35%; *P*=.07) and observed no increased adverse outcomes in the eConsult group. Together, these findings suggest that surgical eConsults maximize the efficiency of surgical care delivery without compromising safety; however, larger studies are required.

#### Health Outcomes of Patients Following an eConsult

Despite the aforementioned benefits of eConsults, patient outcomes were marginally improved, as evidenced in medical subspecialties [78,79]. There is hope that surgical eConsults will decrease the rate of emergency department presentations during the increasingly lengthy wait period for in-person evaluation; however, this is yet to be studied.

#### Role of Surgical eConsults in the COVID-19 Era

We could only identify 3% (1/33) of studies on surgical eConsults that addressed issues during the COVID-19 pandemic [54], whereas there were reported increases in the number of medical eConsults in the same period [30,80]. One of these studies found a trend of increased eConsult use by PCPs, and the rate of subsequent face-to-face referrals also increased. The latter paradoxical finding requires exploration in surgical subspecialties, as it may reflect eConsults being used for different patient presentations compared with before the pandemic. At our service, we have an increasingly large backlog of surgical outpatients because of the pandemic who would benefit from virtual specialist advice even in the absence of face-to-face appointments. Furthermore, elective surgery cancellations have increased operation wait times, and these patients could be more efficiently optimized virtually via eConsults rather than attending a face-to-face preadmission clinic.

### Enhancing Patient Experience of Care

#### Patient Satisfaction With Surgical eConsults

Only 6% (2/33) of studies directly assessed patient satisfaction with surgical eConsults and found that a minority (6% (1/17) and 19% (65/342), respectively) of patients preferred the traditional referral pathway [11,53]. Reservations about eConsults were addressed in some services by allowing for a section in the eConsult where patient preference for a face-to-face consultation can be noted [44]. There also remain many questions regarding the patient’s right to access the surgeon’s response. When the eConsult information is added to the patient’s EMR, their access rights may be governed by laws of freedom of information; however, when a private or outsourced eConsult platform is used, disclosure of information may not be straightforward. State-based legislation must keep pace with eConsult uptake to ensure that patients can gain equitable access to their eConsult. The paucity of firsthand data for patient experience is because of the convenience of using retrospective analysis of PCP exit surveys, which most studies used. In these studies, PCPs thought that 93% to 94.4% of surgical eConsults had good or excellent value for their patients [50,60,63].

#### Rate of Avoided Face-to-face Consultations Because of Surgical eConsults

In *optional-pathway* eConsult services, the rate of face-to-face visits following eConsults was 5.4% to 36% [11,18,43,56,57,65]. The rate was higher for *single-pathway* eConsult services, at 37% to 92.6%, as there is no triage process for low-acuity conditions [44,64,66,67]. The rates of face-to-face follow-up after eConsults were similar between surgical and medical specialties [43,44], suggesting that surgical conditions do not require in-person evaluation more frequently despite the interventional nature of the specialty. Surgical conditions with low rates of face-to-face follow-up or high requirements for prereferral investigations benefit more from eConsults than conditions that are immediately scheduled for an in-person visit with no additional workup (ie, the traditional referral pathway). For example, in a retrospective study of 472 urology eConsults, Chertack et al [68] found that only 23% of patients referred for renal cysts required a face-to-face consultation compared with 80% (24/30) of patients with an elevated prostate-specific antigen (PSA), confirmed in another study (89% (42/47) of elevated PSA cases and after further workup in 11% (5/47) of cases [67]). One could speculate that raised PSA frequently necessitates shared decision-making, requiring an in-person visit. Services with specific criteria for which an eConsult can take place or those with dedicated triage clinicians [61,65] can increase the rates of resolved eConsults, with 3% (1/33) of the studies showing no cases of face-to-face follow-up when eConsults were triaged appropriately [61]. Similarly, some subspecialties still require a reasonable percentage of face-to-face visits, ranging from 90.2% (1761/1952) in otolaryngology to 71.6% (277/387) in obstetrics in one of the studies [44]. This may be a reflection of the variability in reliance on physical examination between subspecialties, with those that lean more heavily on imaging requiring fewer face-to-face visits.

There is a glaring lack of data regarding the underlying characteristics of *resolved* eConsults (ie, not scheduled for a face-to-face visit)—why some surgical conditions, subspecialties, or eConsult questions are more or less likely to avoid a face-to-face visit. Furthermore, each surgical subspecialty sees a diverse range of conditions, and broad generalizations about the viability of each subspecialty for eConsults would belie the heterogeneity of the patient population. In addition, generalizations cannot be made about which subspecialties benefit more from low rates of subsequent face-to-face referral in *optional-pathway* eConsult services, as this will vary according to the eligibility criteria for eConsults.

Studies on *single-pathway* eConsults defined avoided unnecessary face-to-face visits as cases where the PCP had contemplated a face-to-face referral but decided not to as a result of the eConsult. eConsults that result in virtual management cannot be included, as PCPs often submit questions via eConsults that they would not have referred as face-to-face consultations. Most studies on surgical eConsults showed them to avoid a face-to-face visit in 33% to 68% of patients [50,52,60,62,64]. Avoidance rates for surgical subspecialties appear similar to those for medical subspecialties. Approximately 6% (2/33) of multispecialty studies found that orthopedics had the highest rate of unnecessary referral avoidance, at 38% (∼62/162) and 55% (∼6/11), respectively, while also showing that otolaryngology had the lowest rate, at 15% (∼4/26) and 8% (∼1/12), respectively [50,60]. A single-specialty orthopedic study found a similar rate of referral avoidance [62], and therefore, it represents a promising subspecialty in which eConsults should play an increasing role. Conversely, single-specialty studies on otolaryngology eConsults have not replicated this low rate of referral avoidance [52], and thus, further studies must be conducted. Reducing avoidable face-to-face visits not only saves patients time and money but is also beneficial to clinicians—Kinberg et al [18] suggested that avoided referrals free up specialists’ time for patients who require a more urgent review. They found that the mean wait time for face-to-face specialist visits decreased from 60.8 to 42.8 days following eConsult implementation. Another study noted similar trends of reduction in elective surgery wait times [27].

#### Time Savings as a Result of Surgical eConsults

Estimates for the time taken for specialist responses ranged from 19.9 hours to 3.6 days [49,50,56,57,59,62,64,68]. The corresponding waiting time via a traditional face-to-face visit ranged from 54 to 482.5 days [50,52,62]. Approximately 6% (2/33) of the studies noted that the time to treatment onset was also shorter with eConsults, showing that time savings probably translate to patient benefit [49,53].

Approximately 6% (2/33) of urological studies noted that eConsults increased the efficiency of patient care by expediting their workup [69,70], in some cases dramatically (eg, Bergman et al [69] reported a decreased time from documented hematuria to completed a workup from 404 to 192 days). This is especially true in surgical subspecialties where a radiological or procedural diagnosis is common and is not usually ordered by the PCP alone.

### Improving the Work Life of Clinicians

The proportion of PCPs who rated surgical eConsults as having good or excellent value for themselves ranged from 87% to 97% [11,50,60,62-64], with an educational benefit in 60% to 89% [44,62]. A critical factor in PCP satisfaction is the quality of the specialist response. Tuot et al [44] was the only study to examine eConsultant competencies and found that a lower referral volume (<900 per year), a physician rather than nurse reviewer, and more time spent per referral (>7 minutes) were associated with higher-quality surgical and medical eConsults, as judged by the PCP. There was hope that PCP education would gradually obviate the need for future eConsults regarding the same issue; however, specialist surveys noted that PCPs often repeat questions [59]. Approximately 3% (1/33) of the studies demonstrated that a feedback session for specialists to improve their eConsult response quality resulted in a significant increase in high-quality eConsult reviews at 3 months [44]—a similar feedback session could be used for PCPs to improve their referral quality, with more frequent sessions being used to sustain long-term benefits.

eConsults undoubtedly alter the relationship between PCPs and specialists; in many cases, eConsults are replacing the informal *curbside* conversation. Although this traditional form of specialist consultation is still widely used, Gupte et al [59], in a survey of PCPs, found that the formal documentation of surgical eConsults was seen as a key drawcard. Indeed, although medicolegal concerns are often cited as an issue with eConsults, the permanent electronic recording of PCP-specialist consultations confers a degree of medicolegal protection when compared with undocumented curbside conversations [52]. Other features associated with eConsult uptake have been studied elsewhere [81]; a pertinent finding is that PCPs with longer practicing time are less likely to submit eConsults, which suggests that familiarity with the curbside system fosters an unwillingness to adopt new methods. It is possible that mandatory eConsults or investigations before face-to-face visits deter veteran PCPs from using eConsults; however, the specific reasons for this trend require further investigation.

Surveys of specialists noted that eConsults freed up face-to-face appointment times, and most indicated that eConsults did not increase their workload [50,59]. However, specialist satisfaction with surgical eConsults has been much more variable and more poorly studied than PCP satisfaction. Most specialists were able to respond to the eConsult within 20 minutes [44,52,56,60,63,64]. Kinberg et al [18] noted a decrease in cancellations or failure to attend face-to-face otolaryngology clinics from 38.9% (1141/2932) to 19.3% (713/3686) after eConsult implementation. One could speculate that the eConsult system allowed for triaging of patients who were anxious to seek in-person evaluation.

Approximately 6% (2/33) of the studies noted an unanticipated use of eConsults by clinicians. Gupte et al [59] found that 73.3% (487/664) of orthopedic eConsults were initiated by an orthopedic clinician using the eConsult system for ease of generating a preoperative chart review. Frequent spot checks to ensure the *PCP* is not the same as the *specialist* should be performed in all eConsult services to ensure this practice is not taking place. Parikh et al [49] noted that neurosurgeons electronically contacted 12% (8/69) of patients directly; however, this may represent a more well-rounded eConsult and does not mean that the guidelines were being deliberately disregarded. Direct patient contact may reduce time delays arising from obtaining patient information secondhand through the PCP and should be encouraged when it is being used to supplement, and not replace, the eConsult. Furthermore, details of contact between the patient and specialist need to be accessible to the PCP to ensure that they remain informed of the patient’s case and maintain their educational benefit.

### Reducing Per Capita Cost of Health Care

All cost-saving analyses found that surgical eConsults were associated with reduced costs to patients and health services [43,50,51,58,60]. Some studies drew similar conclusions on indirect outcomes, such as differences in specialist payments [53,54]. Approximately 6% (2/33) of the studies found that savings were because of reduced outpatient rather than inpatient costs, possibly from a reduction in diagnostic tests and procedures and more rapid initiation of treatment [51,58]. Anderson et al [51] also found that health service cost savings were greater in orthopedics than in the 3 medical specialties, possibly because orthopedic visits involve more costly procedures, which yield greater savings when avoided.

The caveat to these results is that all studies were limited in their ability to estimate cost savings because of the large number of variables that cannot be accounted for, such as costs incurred outside a given health service. Furthermore, the longest comprehensive cost analysis was 3 years.

## Discussion

### Principal Findings

**Figure 4:**
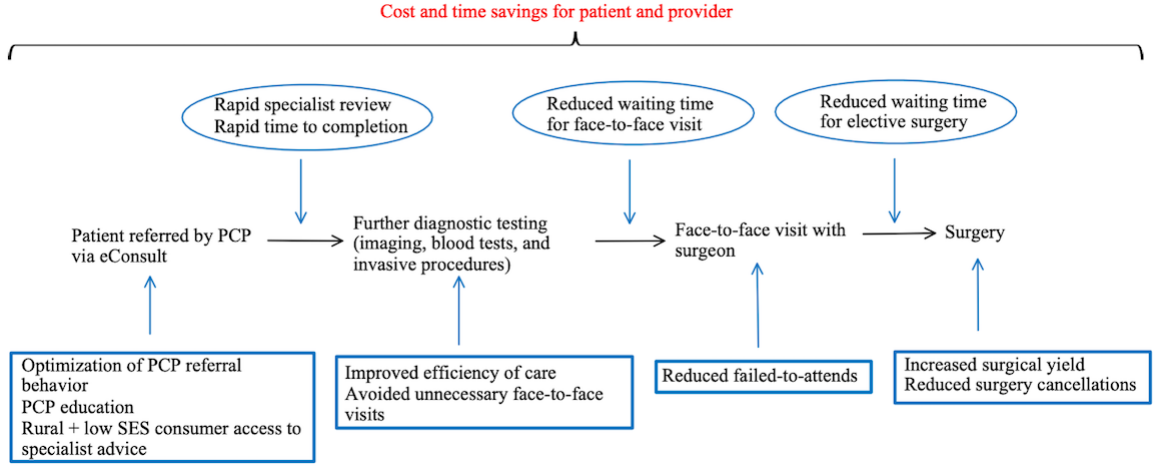
A schematic representation of some of the benefits of surgical electronic consultations (eConsults). PCP: primary care provider; SES: socioeconomic status.

A summary of the findings of this review within the Quadruple Aim Framework (Table 2 and Figure 4) shows that surgical eConsults have benefits at every step of the referral pathway. We found that surgical eConsults showed significant benefits in time and cost savings, reducing surgical outpatient wait times and increasing access to surgical care in underserved patient populations. Although the uptake in surgical subspecialties has been less enthusiastic than in medical subspecialties, the broad outcomes are similar in the 2 fields. Although many concerns common to medical and surgical eConsults (such as workload increases, medicolegal protection, and reimbursement) were found to be unfounded or surmountable, specific surgical concerns (eg, the erosion of the patient-surgeon relationship before surgery) could still be addressed.

**Table 2:** Summary of the benefits, limitations, and future work for surgical electronic consultations (eConsults) within the Quadruple Aim Framework.

Category	Benefits	Limitations	Future work
Improving population health	Safety comparable with traditional referral systems Increased or equal surgical yield Yields a new or confirmed course of PCP^a^ management Alterations in PCP referral behavior	Frequent lack of contingency for rereferral Frequent lack of documentation of PCP follow-up Clinicians with higher volumes of eConsults spend less time per eConsult	Randomized studies to assess long-term patient outcomes Long-term studies on safety Change in eConsult use since the COVID-19 pandemic
Enhancing the patient experience of care	Decreased wait time for a surgical opinion Increased efficiency of care Avoidance of unnecessary face-to-face consultations Drive time savings Decreased wait time for face-to-face and elective surgery Decreased unnecessary invasive investigations	Impersonal nature of eConsults Patient privacy issues Common patient preference for face-to-face consultations	Large-scale surveys of patient satisfaction Identification of viable conditions for eConsults in each surgical subspecialty Empirical evaluation of eConsults in expediting patient workup
Improving the work life of clinicians	High PCP satisfaction PCP education Reduction in failed-to-attend consultations	Medicolegal ramifications Technological limitations Difficulties with eConsults from external health services Increased specialist workload Inappropriate and incomplete referrals Repetitive questions from PCPs Variability in eConsult delivery platforms	Large-scale surveys of specialist satisfaction Studies on factors associated with high-quality eConsults Studies assessing the prevalence of specialist-patient communication in eConsults
Reducing per capita cost of health care	Cost savings to patient and health service	Funding model implementation concerns Insufficient specialist reimbursement	Confirmation of reduced unnecessary diagnostic procedures with eConsults Long-term studies on cost savings

^a^PCP: primary care provider.

### Comparison With Prior Work

This paper builds on 2 recent systematic reviews of combined medical and surgical eConsults [39,40,82,83]. The 2019 review by Vimalananda et al [39] included 63 studies in their analysis, most of which were observational, which is similar to our findings. They were able to identify 2 studies on medical subspecialties (nephrology and endocrinology) that compared the clinical outcomes of eConsult patients with face-to-face referrals, whereas we could identify no such studies on patients of surgery. Liddy et al [40] included 43 studies in their analysis and were notably able to show cost savings because of eConsult use. Our finding on cost savings is also consistent with a scoping review that suggested target key areas where money can be saved using telehealth (eg, mitigating the need for expensive, unnecessary procedures) [84]. This and our overall findings should be reassuring to health services looking to implement surgical eConsults into their workflow. The most common barriers to eConsult implementation that we identified—namely, medicolegal, workload, and reimbursement concerns—are very similar to recent articles dedicated to this topic [81,85-88] and, therefore, were not discussed in this paper.

### Limitations of This Review

Limitations include that the studies were from a limited cohort from 3 well-established eConsult services, and only 3% (1/33) of the studies were performed in a low socioeconomic status country [71]. There is a significant overrepresentation of some subspecialties, and all data were observational, which raises the possibility of unknown factors causing the outcomes described in this review. Randomization has been used in medical eConsult studies [89,90]; however, this may not be reflective of real-world conditions. At this stage, it would be advisable for further work to shift its focus from well-established markers of eConsult benefits to analyzing pitfalls and safety concerns. We did not conduct individual appraisals of study quality. Furthermore, it was impossible to include all the multispecialty studies where surgery was a subanalysis of the broader population, given that our search strategy missed studies that did not include a surgical term in the title or abstract. Our exclusion of non-English articles may also have missed other studies from low socioeconomic status countries. Concerns such as medicolegal issues and management responsibilities were raised in the discussion sections of some papers but could not be included in the data.

### Future Directions

There are some important understudied components of surgical eConsults. An example is the effect of differing patient-surgeon relationships before a major operation on patient satisfaction with a virtual platform. Furthermore, we identified factors associated with a high-quality eConsult response, and this can be leveraged in future work that can explore these features, such as prior telemedicine training, to optimize the quality of responses. Other work can confirm the economical use of diagnostic investigations within the eConsult system. Finally, assessment of eConsult outcomes in specific surgical subspecialties (eg, cardiothoracic surgery) may show benefits associated with eConsults for a specific specialty.

### Conclusions

In conclusion, eConsults represent a safe and advantageous alternative to face-to-face consultations in surgical clinics. Specific surgical subspecialties and conditions appear to benefit more from eConsults, although, even in cases where an in-person visit is needed, eConsults serve to expedite the patient’s workup. For most outcomes, surgical eConsults performed similarly to medical eConsults. Most limitations of surgical eConsults are system-level issues that can be addressed by appropriate implementation protocols, including clinician training and automatic safeguards. Future work on surgical eConsults should further elucidate long-term safety considerations, patient perspectives, and the effects of evolving practices during the COVID-19 pandemic.

## Appendix

Multimedia Appendix 1 Search strategy.

Multimedia Appendix 2 Summary of the included studies.

## Abbreviations

eConsult: electronic consultation
EMR: electronic medical record
eReferral: electronic referral
PCP: primary care provider
PRISMA: Preferred Reporting Items for Systematic Reviews and Meta-Analyses
PSA: prostate-specific antigen

## References

[1] Gadzinski AJ, Gore JL, Ellimoottil C, Odisho AY, Watts KL (2020). Implementing telemedicine in response to the COVID-19 pandemic. J Urol.

[2] Snoswell CL, Caffery LJ, Haydon HM, Thomas EE, Smith AC (2020). Telehealth uptake in general practice as a result of the coronavirus (COVID-19) pandemic. Aust Health Rev.

[3] Leow AH, Mahadeva S (2021). Telemedicine in routine gastroenterology practice: a boost during the COVID-19 pandemic. JGH Open.

[4] McMaster T, Wright T, Mori K, Stelmach W, To H (2021). Current and future use of telemedicine in surgical clinics during and beyond COVID-19: a narrative review. Ann Med Surg (Lond).

[5] Rimmer RA, Christopher V, Falck A, de Azevedo Pribitkin E, Curry JM, Luginbuhl AJ, Cognetti DM (2018). Telemedicine in otolaryngology outpatient setting-single Center Head and Neck Surgery experience. Laryngoscope.

[6] Stewart C, Coffey-Sandoval J, Reid MW, Ho TC, Lee TC, Nallasamy S (2021). Reliability of telemedicine for real-time paediatric ophthalmology consultations. Br J Ophthalmol (forthcoming).

[7] Debono B, Bousquet P, Sabatier P, Plas JY, Lescure JP, Hamel O (2016). Postoperative monitoring with a mobile application after ambulatory lumbar discectomy: an effective tool for spine surgeons. Eur Spine J.

[8] DeAntonio JH, Kang HS, Cockrell HC, Rothstein W, Oiticica C, Lanning DA (2019). Utilization of a handheld telemedicine device in postoperative pediatric surgical care. J Pediatr Surg.

[9] Ignatowicz A, Atherton H, Bernstein CJ, Bryce C, Court R, Sturt J, Griffiths F (2019). Internet videoconferencing for patient-clinician consultations in long-term conditions: a review of reviews and applications in line with guidelines and recommendations. Digit Health.

[10] Davarpanah AH, Mahdavi A, Sabri A, Langroudi TF, Kahkouee S, Haseli S, Kazemi MA, Mehrian P, Mahdavi A, Falahati F, Tuchayi AM, Bakhshayeshkaram M, Taheri MS (2020). Novel screening and triage strategy in Iran during deadly coronavirus disease 2019 (COVID-19) epidemic: value of humanitarian teleconsultation service. J Am Coll Radiol.

[11] Chittle MD, Rao SK, Jaff MR, Patel VI, Gallen KM, Avadhani R, Ferris TG, Wasfy JH (2015). Asynchronous vascular consultation via electronic methods: a feasibility pilot. Vasc Med.

[12] Tuot DS, Liddy C, Vimalananda VG, Pecina J, Murphy EJ, Keely E, Simon SR, North F, Orlander JD, Chen AH (2018). Evaluating diverse electronic consultation programs with a common framework. BMC Health Serv Res.

[13] Joschko J, Keely E, Grant R, Moroz I, Graveline M, Drimer N, Liddy C (2018). Electronic consultation services worldwide: environmental scan. J Med Internet Res.

[14] Barnett ML, Yee HF, Mehrotra A, Giboney P (2017). Los Angeles Safety-Net Program eConsult system was rapidly adopted and decreased wait times to see specialists. Health Aff (Millwood).

[15] Chen AH, Murphy EJ, Yee HF (2013). eReferral--a new model for integrated care. N Engl J Med.

[16] Gleason N, Ackerman S, Shipman SA (2018). eConsult-transforming primary care or exacerbating clinician burnout?. JAMA Intern Med.

[17] Lee J, Rikin S, Jain R (2021). Identifying content themes in primary care physician and rheumatologist communications within electronic consultations: a qualitative study. ACR Open Rheumatol.

[18] Kinberg EC, Kirke DN, Trosman SJ (2021). Modernizing the otolaryngology referral workflow: the impact of electronic consultation. Laryngoscope.

[19] Zuchowski JL, Rose DE, Hamilton AB, Stockdale SE, Meredith LS, Yano EM, Rubenstein LV, Cordasco KM (2015). Challenges in referral communication between VHA primary care and specialty care. J Gen Intern Med.

[20] Cyr ME, Etchin AG, Guthrie BJ, Benneyan JC (2019). Access to specialty healthcare in urban versus rural US populations: a systematic literature review. BMC Health Serv Res.

[21] Mayer ML, Skinner AC, Slifkin RT, National Survey of Children With Special Health Care Needs (2004). Unmet need for routine and specialty care: data from the National Survey of Children With Special Health Care Needs. Pediatrics.

[22] Kuhlthau K, Nyman RM, Ferris TG, Beal AC, Perrin JM (2004). Correlates of use of specialty care. Pediatrics.

[23] Kuhlthau K, Ferris TG, Beal AC, Gortmaker SL, Perrin JM (2001). Who cares for medicaid-enrolled children with chronic conditions?. Pediatrics.

[24] Cook NL, Hicks LS, O'Malley AJ, Keegan T, Guadagnoli E, Landon BE (2007). Access to specialty care and medical services in community health centers. Health Aff (Millwood).

[25] Hofstetter PJ, Kokesh J, Ferguson AS, Hood LJ (2010). The impact of telehealth on wait time for ENT specialty care. Telemed J E Health.

[26] Leddin D, Bridges RJ, Morgan DG, Fallone C, Render C, Plourde V, Gray J, Switzer C, McHattie J, Singh H, Walli E, Murray I, Nestel A, Sinclair P, Chen Y, Irvine EJ (2010). Survey of access to gastroenterology in Canada: the SAGE wait times program. Can J Gastroenterol.

[27] Valsangkar NP, Eppstein AC, Lawson RA, Taylor AN (2017). Effect of lean processes on surgical wait times and efficiency in a tertiary care Veterans Affairs medical center. JAMA Surg.

[28] Gadzinski AJ, Andino JJ, Odisho AY, Watts KL, Gore JL, Ellimoottil C (2020). Telemedicine and eConsults for hospitalized patients during COVID-19. Urology.

[29] Rikin S, Epstein EJ, Gendlina I (2021). Rapid implementation of inpatient eConsult programme addresses new challenges for patient care during COVID-19 pandemic. BMJ Innov.

[30] Leyton C, Zhang C, Rikin S (2022). Evaluation of the effects of the COVID-19 pandemic on electronic consultation use in primary care. Telemed J E Health.

[31] Phadke NA, Del Carmen MG, Goldstein SA, Vagle J, Hidrue MK, Botti ES, Wasfy JH (2020). Trends in ambulatory electronic consultations during the COVID-19 pandemic. J Gen Intern Med.

[32] Tran CS, Liddy CE, Liu DM, Afkham A, Keely EJ (2016). eConsults to endocrinologists improve access and change primary care provider behavior. Endocr Pract.

[33] Anderson D, Villagra V, Coman EN, Zlateva I, Hutchinson A, Villagra J, Olayiwola JN (2018). A cost-effectiveness analysis of cardiology eConsults for Medicaid patients. Am J Manag Care.

[34] Kim EJ, Orlander JD, Afable M, Pawar S, Cutrona SL, Simon SR, Strymish J, Vimalananda VG (2019). Cardiology electronic consultation (e-consult) use by primary care providers at VA medical centres in New England. J Telemed Telecare.

[35] Bradi AC, Sitwell L, Liddy C, Afkham A, Keely E (2018). Ask a neurologist: what primary care providers ask, and reducing referrals through eConsults. Neurol Clin Pract.

[36] Keely E, Li J, Magner P, Afkham A, Liddy C (2018). Nephrology eConsults for primary care providers: original investigation. Can J Kidney Health Dis.

[37] Fulford D, Tuot DS, Mangurian C (2016). Electronic psychiatric consultation in primary care in the safety net. Psychiatr Serv.

[38] Venkatesh RD, Campbell EJ, Thiim M, Rao SK, Ferris TG, Wasfy JH, Richter JM (2019). e-Consults in gastroenterology: an opportunity for innovative care. J Telemed Telecare.

[39] Vimalananda VG, Orlander JD, Afable MK, Fincke BG, Solch AK, Rinne ST, Kim EJ, Cutrona SL, Thomas DD, Strymish JL, Simon SR (2020). Electronic consultations (E-consults) and their outcomes: a systematic review. J Am Med Inform Assoc.

[40] Liddy C, Moroz I, Mihan A, Nawar N, Keely E (2019). A systematic review of asynchronous, provider-to-provider, electronic consultation services to improve access to specialty care available worldwide. Telemed J E Health.

[41] Mold F, Hendy J, Lai YL, de Lusignan S (2019). Electronic consultation in primary care between providers and patients: systematic review. JMIR Med Inform.

[42] To H, McMaster T, Stelmach W (2021). Addressing telemedicine challenges for surgery clinics in the Post-COVID era. ANZ J Surg.

[43] Saxon DR, Kaboli PJ, Haraldsson B, Wilson C, Ohl M, Augustine MR (2021). Growth of electronic consultations in the Veterans Health Administration. Am J Manag Care.

[44] Tuot DS, Murphy EJ, McCulloch CE, Leeds K, Chan E, Chen AH (2015). Leveraging an electronic referral system to build a medical neighborhood. Healthc (Amst).

[45] Bodenheimer T, Sinsky C (2014). From triple to quadruple aim: care of the patient requires care of the provider. Ann Fam Med.

[46] Liddy C, Keely E (2018). Using the quadruple aim framework to measure impact of heath technology implementation: a case study of eConsult. J Am Board Fam Med.

[47] Osman MA, Schick-Makaroff K, Thompson S, Bialy L, Featherstone R, Kurzawa J, Zaidi D, Okpechi I, Habib S, Shojai S, Jindal K, Braam B, Keely E, Liddy C, Manns B, Tonelli M, Hemmelgarn B, Klarenbach S, Bello AK (2019). Barriers and facilitators for implementation of electronic consultations (eConsult) to enhance access to specialist care: a scoping review. BMJ Glob Health.

[48] Barnett-Page E, Thomas J (2009). Methods for the synthesis of qualitative research: a critical review. BMC Med Res Methodol.

[49] Parikh PJ, Mowrey C, Gallimore J, Harrell S, Burke B (2017). Evaluating e-consultation implementations based on use and time-line across various specialties. Int J Med Inform.

[50] Lai L, Liddy C, Keely E, Afkham A, Kurzawa J, Abdeen N, Audcent T, Bromwich M, Brophy J, Carsen S, Fournier A, Fraser-Roberts L, Gandy H, Hui C, Johnston D, Keely K, Kontio K, Lamontagne C, Major N, O'Connor M, Radhakrishnan D, Reisman J, Robb M, Samson L, Sell E, Splinter W, van Stralen J, Venkateswaran S, Murto K (2018). The impact of electronic consultation on a Canadian tertiary care pediatric specialty referral system: a prospective single-center observational study. PLoS One.

[51] Anderson D, Villagra VG, Coman E, Ahmed T, Porto A, Jepeal N, Maci G, Teevan B (2018). Reduced cost of specialty care using electronic consultations for Medicaid patients. Health Aff (Millwood).

[52] Kohlert S, Murphy P, Tse D, Liddy C, Afkham A, Keely E (2018). Improving access to otolaryngology-head and neck surgery expert advice through eConsultations. Laryngoscope.

[53] Salazar-Fernandez CI, Herce J, Garcia-Palma A, Delgado J, Martín JF, Soto T (2012). Telemedicine as an effective tool for the management of temporomandibular joint disorders. J Oral Maxillofac Surg.

[54] Corbetta-Rastelli CM, Morgan TK, Homaifar N, Deangelis L, Autry AM (2021). Experiences in electronic consultation (eConsult) service in gynecology from a quaternary academic medical center. J Med Syst.

[55] Afable MK, Gupte G, Simon SR, Shanahan J, Vimalananda V, Kim EJ, Strymish J, Orlander JD (2018). Innovative use of electronic consultations in preoperative anesthesiology evaluation at VA Medical Centers in New England. Health Aff (Millwood).

[56] Patel M, Gadzinski AJ, Bell AM, Watts K, Steppe E, Odisho AY, Yang CC, Ellimoottil C (2021). Interprofessional consultations (eConsults) in urology. Urol Pract.

[57] Gilani S, Bommakanti K, Friedman L (2020). Electronic consults in otolaryngology: a pilot study to evaluate the use, content, and outcomes in an academic health system. Ann Otol Rhinol Laryngol.

[58] Whittington MD, Ho PM, Kirsh SR, Kenney RR, Todd-Stenberg J, Au DH, Simonetti J (2021). Cost savings associated with electronic specialty consultations. Am J Manag Care.

[59] Gupte G, Vimalananda V, Simon SR, DeVito K, Clark J, Orlander JD (2016). Disruptive innovation: implementation of electronic consultations in a Veterans Affairs health care system. JMIR Med Inform.

[60] Liddy C, McKellips F, Armstrong CD, Afkham A, Fraser-Roberts L, Keely E (2017). Improving access to specialists in remote communities: a cross-sectional study and cost analysis of the use of eConsult in Nunavut. Int J Circumpolar Health.

[61] Mann R, van de Weijer PH (2018). Adopting innovation in gynaecology: the introduction of e-consult. Aust N Z J Obstet Gynaecol.

[62] Chang Y, Carsen S, Keely E, Liddy C, Kontio K, Smit K (2020). Electronic consultation systems: impact on pediatric orthopaedic care. J Pediatr Orthop.

[63] Shehata F, Posner G, Afkham A, Liddy C, Keely E (2016). Evaluation of an electronic consultation service in obstetrics and gynecology in Ontario. Obstet Gynecol.

[64] Witherspoon L, Liddy C, Afkham A, Keely E, Mahoney J (2017). Improving access to urologists through an electronic consultation service. Can Urol Assoc J.

[65] Castaneda PR, Duffy B, Andraska EA, Stevens J, Reschke K, Osborne N, Henke PK (2020). Outcomes and safety of electronic consult use in vascular surgery. J Vasc Surg.

[66] Ulloa JG, Russell MD, Chen AH, Tuot DS (2017). A cohort study of a general surgery electronic consultation system: safety implications and impact on surgical yield. BMC Health Serv Res.

[67] McGeady JB, Blaschko SD, Brajtbord JS, Sewell JL, Chen AH, Breyer BN (2014). Electronic preconsultation as a method of quality improvement for urological referrals. Urology Practice.

[68] Chertack N, Lotan Y, Mayorga C, Mauck R (2020). Implementation of a urology e-consult service at a safety net county hospital. Urology Practice.

[69] Bergman J, Neuhausen K, Chamie K, Scales CD, Carter S, Kwan L, Lerman SE, Aronson W, Litwin MS (2013). Building a medical neighborhood in the safety net: an innovative technology improves hematuria workups. Urology.

[70] Pannell SC, Soni SM, Giboney P, Santamaria A, Bergman J (2019). Access to urologic care through clinical integration in a large, underserved population. JAMA Surg.

[71] Olayiwola JN, Udenyi ED, Yusuf G, Magaña C, Patel R, Duck B, Sajanlal S, Potapov A, Kibuka C (2020). Leveraging electronic consultations to address severe subspecialty care access gaps in Nigeria. J Natl Med Assoc.

[72] Kim-Hwang JE, Chen AH, Bell DS, Guzman D, Yee HF, Kushel MB (2010). Evaluating electronic referrals for specialty care at a public hospital. J Gen Intern Med.

[73] Castaneda P, Ellimoottil C (2020). Current use of telehealth in urology: a review. World J Urol.

[74] Wiadji E, Mackenzie L, Reeder P, Gani JS, Carroll R, Smith S, Frydenberg M, O'Neill CJ (2021). Utilization of telehealth by surgeons during the COVID 19 pandemic in Australia: lessons learnt. ANZ J Surg.

[75] Chen AH, Kushel MB, Grumbach K, Yee HF (2010). Practice profile. A safety-net system gains efficiencies through 'eReferrals' to specialists. Health Aff (Millwood).

[76] Brooke BS, Stone DH, Cronenwett JL, Nolan B, DeMartino RR, MacKenzie TA, Goodman DC, Goodney PP (2014). Early primary care provider follow-up and readmission after high-risk surgery. JAMA Surg.

[77] Kripalani S, LeFevre F, Phillips CO, Williams MV, Basaviah P, Baker DW (2007). Deficits in communication and information transfer between hospital-based and primary care physicians: implications for patient safety and continuity of care. JAMA.

[78] Oseran AS, Wasfy JH (2019). Early experiences with cardiology electronic consults: a systematic review. Am Heart J.

[79] Patel PS, Jiang B, Marcelli M, Mediwala SN, Vasudevan MM (2019). Electronic consultation: an effective alternative to in-person clinical care for patients with diabetes mellitus. J Diabetes Sci Technol.

[80] Greiwe J (2020). Telemedicine in a post-COVID world: how eConsults can be used to augment an allergy practice. J Allergy Clin Immunol Pract.

[81] Bilodeau H, Deri Armstrong CD, Keely E, Liddy C (2018). Who uses eConsult? investigating physician characteristics associated with usage (and nonusage). Telemed J E Health.

[82] Vimalananda VG, Gupte G, Seraj SM, Orlander J, Berlowitz D, Fincke BG, Simon SR (2015). Electronic consultations (e-consults) to improve access to specialty care: a systematic review and narrative synthesis. J Telemed Telecare.

[83] Liddy C, Drosinis P, Keely E (2016). Electronic consultation systems: worldwide prevalence and their impact on patient care-a systematic review. Fam Pract.

[84] Snoswell CL, Taylor ML, Comans TA, Smith AC, Gray LC, Caffery LJ (2020). Determining if telehealth can reduce health system costs: scoping review. J Med Internet Res.

[85] Baines R, Tredinnick-Rowe J, Jones R, Chatterjee A (2020). Barriers and enablers in implementing electronic consultations in primary care: scoping review. J Med Internet Res.

[86] Knox M, Murphy EJ, Leslie T, Wick R, Tuot DS (2020). e-Consult implementation success: lessons from 5 county-based delivery systems. Am J Manag Care.

[87] Liddy C, de Man G, Moroz I, Afkham A, Mercer J, Keely E (2020). Effective integration of an eConsult service into an existing referral workflow within a primary care clinic. Telemed J E Health.

[88] Moroz I, Archibald D, Breton M, Cote-Boileau E, Crowe L, Horsley T, Hyseni L, Johar G, Keely E, Burns KK, Kuziemsky C, Laplante J, Mihan A, Oppenheimer L, Sturge D, Tuot DS, Liddy C (2020). Key factors for national spread and scale-up of an eConsult innovation. Health Res Policy Syst.

[89] Katz IJ, Pirabhahar S, Williamson P, Raghunath V, Brennan F, O'Sullivan A, Youssef G, Lane C, Jacobson G, Feldman P, Kelly J (2018). iConnect CKD - virtual medical consulting: a web-based chronic kidney disease, hypertension and diabetes integrated care program. Nephrology (Carlton).

[90] Olayiwola JN, Anderson D, Jepeal N, Aseltine R, Pickett C, Yan J, Zlateva I (2016). Electronic consultations to improve the primary care-specialty care interface for cardiology in the medically underserved: a cluster-randomized controlled trial. Ann Fam Med.

