# Supplementary material for: The Use of Electronic Consultations in Outpatient Surgery Clinics: Synthesized Narrative Review
Source: JMIR Perioper Med. 2022 Apr 14;5(1):e34661. doi: 10.2196/34661 (PMC9052035; doi:10.2196/34661)
Supplement: Multimedia Appendix 1 [file periop_v5i1e34661_app1.docx]

Multimedia Appendix 1. Search strategy.

| Journal | Search | Results (n) |
| --- | --- | --- |
| OVID Medline | 1. e-consult* or econsult* or electronic consult* or ereferral* or electronic refer* or e consult* or e referral* or electronic communication* or interprofessional consult* or "store and forward" | 2985 |
|  | 2. surg* or general surg* or neurosurg* or obstetric* or gynaecolog* or gynecolog* or cardiothoracic surg* or plastic surg* or urolog* or orthopaedic* or orthopedic* | 3,651,549 |
|  | 3. 1 AND 2 | 279 |
|  | 4. 1 AND 2 - limited to 2011–2021 - English only | 184 |
| OVID EMBASE | 1. e-consult* or econsult* or electronic consult* or ereferral* or electronic refer* or e consult* or e referral* or electronic communication* or interprofessional consult* or "store and forward" | 3954 |
|  | 2. surg* or general surg* or neurosurg* or obstetric* or gynaecolog* or gynecolog* or cardiothoracic surg* or plastic surg* or urolog* or orthopaedic* or orthopedic* | 5,137,164 |
|  | 3. 1 AND 2 | 474 |
|  | 4. 1 AND 2 - English only  - Limited to 2011–2021 - Article, article in press, or review - Exclude Medline journals | 26 |
| SCOPUS | 1. e-consult* or econsult* or "electronic consult*" or ereferral* or "electronic refer*" or "e consult*" or "e referral*" or "electronic communication*" or "interprofessional consult*" | 7297 |
|  | 2. surg* or "general surg*" or neurosurg* or obstetric* or gynaecolog* or gynecolog* or "cardiothoracic surg"* or "plastic surg*" or urolog* or orthopaedic* or orthopedic* | 265,892 |
|  | 3. 1 AND 2 | 29 |
|  | 4. 1 AND 2 - Limited to 2011–2021 - English only - Articles and reviews | 133 |
| Web of science | 1. e-consult* or econsult* or "electronic consult*" or ereferral* or "electronic refer*" or "e consult*" or "e referral*" or "electronic communication*" or "interprofessional consult*" or "store and forward" | 9400 |
|  | 2. surg* or "general surg*" or neurosurg* or obstetric* or gynaecolog* or gynecolog* or "cardiothoracic surg*" or "plastic surg*" or urolog* or orthopaedic* or orthopedic* | 5,023,283 |
|  | 3. 1 AND 2 | 462 |
|  | 4. 1 AND 2 - Limited to 2011–2021 - Articles and review articles - English only | 278 |
