# Supplementary material for: The Use of Electronic Consultations in Outpatient Surgery Clinics: Synthesized Narrative Review
Source: JMIR Perioper Med. 2022 Apr 14;5(1):e34661. doi: 10.2196/34661 (PMC9052035; doi:10.2196/34661)
Supplement: Multimedia Appendix 2 [file periop_v5i1e34661_app2.docx]

Multimedia Appendix 2. Summary of the included studies.

| Author | Year, country | Name of journal | Journal type | Study design | Surgical subspecialty | Sample size (number of surgical eConsults) | Objective | Outcome | Keywords |
| --- | --- | --- | --- | --- | --- | --- | --- | --- | --- |
|  |  |  |  |  |  |  |  |  |  |
| Afable et al. | 2018 USA | Health Affairs | Health services | Mixed methods - retrospective audit + survey | Anaesthesia | 7988 | Quality improvement project to assess variability of eConsult use across anaesthetics departments in the Veterans' Affairs New England Healthcare System (VANEHS) + clinicians' perceptions of eConsults | eConsult use had no significant effect on preventable surgery cancellation rates. Clinicians felt eConsult simplified the preoperative process, rural access was facilitated and time was saved, but were impersonal. | Information technology, organization, delivery of care |
| Anderson et al. | 2018 USA | Health Affairs | Health Services | Retrospective audit | Orthopaedic surgery | 77 | Assess the cost-effectiveness of eConsults in Medicaid patients | eConsults decrease the number of face-to-face visits required without change to total referral volume. eConsults decrease the average monthly costs per patient, likely due to saving in diagnostic tests and procedures with eConsults. | Nil |
| Bergman et al. | 2013 USA | Urology | Surgical | Retrospective audit | Urology | 50 | Examine the impact of the eReferral system on the time for hematuria workup | eConsults expedited the workup of haematuria and decreased the time to upper urinary tract imaging. | Nil |
| Castaneda and Ellimootil | 2020 USA | World Journal of Urology | Surgical | Systematic review | Urology | N/A | Review the current applications of telehealth in urology | eConsults are beneficial for low-acuity patients and enhance interdisciplinary patient care. They are limited by inexperience with new technologies, changing staff roles, issues with licencing, and reimbursement | Telemedicine, telehealth, patient-centred care, health communication |
| Castaneda et al. | 2020 USA | Journal of Vascular Surgery | Surgical | Retrospective audit | Vascular surgery | 350 | Determine the use of eConsults in vascular surgery in a Veterans Affairs (VA) context and the safety of their use | eConsults were a safe method of assessment of low-risk conditions when compared to face-to-face consultations. Only a very small percentage of low-risk vascular surgery conditions require a subsequent face-to-face appointment | eConsult, access to care, communication, coordination of care |
| Chang et al. | 2020 Canada | Journal of Pediatric Orthopedics | Surgical | Mixed methods - retrospective audit + survey | Paediatric orthopaedic surgery | 212 | Analyse the impact of eConsults in paediatric orthopaedics | eConsults are highly acceptable to PCPs. eConsults results in avoidance of face-to-face appointments and altering of PCP referral plans. PCPs suggested that manual uploading of patient data can be difficult and a shared virtual workspace is preferable. | Electronic consultation, paediatric orthopaedics, health care, quality improvement |
| Chertack et al. | 2020 USA | Urology Practice | Surgical | Retrospective audit | Urology | 472 | Assess the implementation of an eConsult service in a urology clinic at a safety net hospital | The majority of eConsults require subsequent face-to-face visits, but this varies widely across presenting complaints. eConsults are associated with rapid specialist response time. | Remote consultation, haematuria, carcinoma, renal cell, prostate-specific antigen |
| Chittle et al. | 2015 USA | Vascular Medicine | Surgical | Retrospective audit | Vascular surgery | 54 | Assess the efficacy and patient and provider satisfaction with an eConsult service | eConsults comprised a minority of total referrals. A small proportion required a face-to-face visit. eConsults were associated with high patient and provider satisfaction. | Accountable Care Organisation (ACO), asynchronous consult, care delivery, chronic disease management, e-consult, vascular care |
| Corbetta-Rastelli et al. | 2021 USA | Journal of Medical Systems | Medical informatics | Retrospective audit | Obstetrics & gynaecology | 548 | Analyse the implementation of the eConsult program in the UCSF gynaecology department | Response to eConsults were rapid. eConsults would result in significant cost savings and were useful to determine if an in-person visit was necessary | Clinical access, consultation, COVID-19, gynaecology, primary care reimbursement, subspecialty services, telehealth, telemedicine |
| Gilani et al. | 2020 USA | Annals of Otology, Rhinology & Laryngology | Surgical | Retrospective audit | Otolaryngology | 64 | Examine reasons for eConsults in otolaryngology and quantify avoidance of face-to-face visits | The majority of otolaryngology eConsults do not require in-person follow-up, and the average specialist response time is fast. Wait time for face-to-face consultation after eConsults is extensive. | eConsult, otolaryngology, primary care, referral, consult |
| Gupte et al. | 2016 USA | JMIR Medical Informatics | Medical informatics | Mixed methods - retrospective audit + survey | Multispecialty | 5141 | Quality improvement study to assess eConsult uptake across the VA healthcare network and clinician acceptability | eConsult use has been adapted for ease of generating a pre-operative chart review via intra-specialty referrals. eConsults arising from other health systems are challenging and are limited by increasing workload, breaks in continuity of care, and repetitive questions by PCPs. PCPs and specialists have high satisfaction with eConsults | Remote consultations, clinical communication, electronic consultation, telehealth, clinical information, decision making, telemonitoring, eHealth infrastructures |
| Kim-Hwang et al. | 2010 USA | Journal of General Internal Medicine | Medicine | Survey | Neurosurgery and orthopaedic surgery | N/A | Examine the differences in eReferrals compared to paper-based referrals | eReferrals were more detailed, more appropriate, and required less follow-up than paper-based referrals. The differences were more pronounced in surgical than medical clinics | Access to care, communication, specialty care |
| Kinberg et al. | 2021 USA | Laryngoscope | Surgical | Retrospective audit | Otolaryngology | 6617 | Examine the impact of eConsults on the otolaryngology clinic at a public hospital | eConsult introduction resulted in decreased rate of face-to-face visits, faster patient evaluation, fewer failed-to-attends, and decrease wait time for a face-to-face appointment. eConsults were limited by the PCP needing to provide sufficient information, and the need to dedicated clinicians to manage referrals | Electronic consultation, clinic access, e-consult |
| Kohlert et al. | 2018 Canada | Laryngoscope | Surgical | Prospective observational cohort study | Otolaryngology | 5597 | Examine the efficacy of eConsults in otolaryngology–head and neck surgery | eConsults were rapidly completed with a quick response time, and altered PCP management in a majority of cases. Nearly half the eConsults patients avoided an unnecessary face-to-face appointment. PCPs reported that eConsults were valuable for them and their patients. There was concern for the medicolegal ramifications of eConsults. | eConsult, telemedicine, telehealth, electronic consultation, eConsultation, wait times |
| Lai et al. | 2018 Canada | PLoS One | General | Prospective observational cohort study | Paediatric surgery | 1064 | Assess whether the use of eConsult in paediatrics has similar advantages to its use in adults | Wait time for specialist opinion was much shorter with eConsults than face-to-face visits. The majority of eConsults resulted in a new course of action, and face-to-face referrals were avoided in many cases. PCPs and specialists were highly satisfied with eConsults, but specialists noted that technology use was a barrier. eConsults resulted in significant cost savings. | Nil |
| Liddy et al. | 2017 Canada | Internal Journal of Circumpolar Health | Medicine | Mixed methods - retrospective audit + survey | Multispecialty | 165* | Examine the trends and assess the efficacy of eConsult use in Nunavut | eConsults are associated with cost and time savings. Surgical eConsults tend to confirm course of action rather than offer new management options when compared to medical eConsults. eConsults results in avoided face-to-face visits. eConsults were seen as valuable for patient and provider. | Primary care, eConsult, electronic consultation, wait times, rural communities, northern communities |
| Liddy et al. | 2019 Canada | Telemedicine Journal and e-Health | Medical informatics | Systematic review | Multispecialty | N/A | Review the impact of eConsults on costs and patient outcomes | Overall reduction in face-to-face visits due to eConsults, with some variation between specialties. eConsults are limited by lack of connectivity between healthcare databases. eConsults have high acceptability to clinicians and patients, however most studies assess patient acceptability via clinical reporting. eConsults are associated with cost savings. | eConsult, telehealth, e-health, telemedicine, technology |
| Mann and ven de Weijer | 2018 New Zealand | ANZ Journal of Obstetrics & Gynecology | Surgical | Retrospective audit | Obstetrics & gynaecology | 1013 | Examine the abilities of an e-consult service in the triaging of PCP referrals in gynaecology | Vast majority of eConsults required no further specialist input at 6 months. A minority of eConsult patients were re-referred for the same compliant within 6 months, with no deaths or hospitalisations | Electronic consultation, general practitioner, gynaecology, primary health care, referral and consultation, specialist medical advice |
| McGeady et al. | 2014 USA | Urology Practice | Surgical | Retrospective audit | Urology | 487 | Determine the efficacy of preconsultative exchange in urology | eConsults increased efficiency of care by enabling diagnostic workup prior to face-to-face visits. eConsults are limited by automatic closure of the referral after 6 months of no activity. | Urology, quality improvement, referral and consultation, primary health care, clinical protocols |
| Olayiwola et al. | 2020 Nigeria | Journal of the National Medical Association | Health services | Mixed methods - retrospective audit + survey | Multispecialty | 23 | Assess the impact of eConsult implementation in Nigeria | eConsult implementation in Nigeria is limited by internet connectivity issues and referral of inappropriately urgent cases. eConsults are highly acceptable to PCPs in Nigeria, and were reported as more useful in medical than surgical subspecialties. | Subspecialty care, access to care, electronic consultation, sub-Saharan Africa, health disparities, telehealth |
| Pannell et al. | 2019 USA | JAMA Surgery | Surgical | Retrospective audit | Urology | 293 | Analyse the ability of eConsults to manage common urologic diagnoses | eConsults improve efficiency of care and are associated with fast completion times. A large proportion can be managed by remote specialist input only. | Nil |
| Parikh et al. | 2017 USA | Internal Journal of Medical Informatics | Medical informatics | Retrospective audit | Neurosurgery | 69 | Examine how eConsults have been implemented across gastroenterology, diabetes, and neurosurgery specialties | A large proportion of neurosurgical eConsults do not require surgery. Far more neurosurgical patients were seen as eConsults than medical patients, which may reflect PCP knowledge about subspecialties. Lack of documentation of PCP follow-up from eConsults is common. Procedure-heavy specialties are associated with longer eConsult referral times. Many neurosurgeons use the eConsult platform inappropriately. | Nil |
| Patel et al. | 2021 USA | Urology Practice | Surgical | Retrospective audit | Urology | 462 | Describe the pattern of usage of urology eConsults and assess their efficacy and value | A minority of eConsults are converted to face-to-face visits, with the most common converted complaint being haematuria. eConsults are associated with a rapid response and short completion time. | Telemedicine, referral, consultation |
| Salazar-Fernandez et al. | 2012 Spain | Journal of Oral and Maxillofacial Surgery | Surgical | Prospective observational cohort study | Oral and maxillofacial surgery | 342 | Determine the efficacy of a store-and-forward telemedicine system for TMJ disorder referrals to an oral and maxillofacial surgery centre | eConsults are associated with rapid treatment onset. eConsults had lower rates of second consults and higher rates of resolved consults than the traditional system. Patients noted anxiety that their concerns were relayed second hand to a specialist, but overall rates of complaints were lower for eConsults. eConsults resulted in fewer lost working hours. | Nil |
| Saxon et al. | 2021 USA | The American Journal of Managed Care | Health services | Retrospective audit | Multispecialty | 3117998* | Outline growth and advantages of eConsults in the VHA | Uptake of surgical eConsults have been growing since at a higher rate than medical specialties. The rate of face-to-face follow-ups is lower in surgical than medical specialties, and has been decreasing. Combined medical and surgical data suggest cost and time savings associated with eConsults. | Nil |
| Shehata et al. | 2016 Canada | Obstetrics & Gynecology | Surgical | Retrospective audit | Obstetrics & gynaecology | 394 | Characterise + assess the effectiveness and useability of the eConsult system in O&G | eConsults alter PCP referral behaviour, facilitate pre-face-to-face workup, and contribute to PCP education. PCPs rate the eConsult service highly. | Nil |
| Tuot et al. | 2015 USA | Healthcare | Health services | Mixed-methods - retrospective audit + prospective study | Multispecialty | 2198* | Identify the usage pattern of eConsults among different specialties and measure the determinants of good-quality referrals and referral feedback efficacy | A minority of eConsults do no require a face-to-face visit at any point. Specialists who spent more time on eConsults are less likely to schedule a face-to-face, and those who have a higher volume of referrals spend less time per referral. The majority of eConsults are considered high-quality by PCPs. The quality of eConsults improves after feedback is given to specialists. | Electronic consultation, medical neighbourhood, primary–specialty interface |
| Ulloa et al. | 2017 USA | BMC Health Services Research | Health services | Retrospective audit | General surgery | 150 | Assess the safety of eConsults and their impact on surgical yield | A minority of eConsults were not scheduled for a face-to-face visit. eConsults improved surgical yield and did not risk patient safety. | Electronic consultation, surgical yield, ambulatory safety, patient-centred medical neighbourhood, health system redesign |
| Valsangkar et al. | 2017 USA | JAMA Surgery | General surgery | Prospective observational cohort study | General surgery | N/A | To assess the impact of the rollout of pilot reforms (including eConsults) in a VA health system | eConsults, among other system-level changes, decreased surgical wait time, increase total operative volume, and decreased failed-to-attend appointments | Nil |
| Vimalananda et al. | 2019 USA | Journal of the American Medical Informatics Association | Medical informatics | Systematic review | Multispecialty | N/A | Summarise the peer reviewed literature on the outcomes of eConsult programs with regard to population health, reducing cost, enhancement of patient and clinical experience | Overall empirical evidence for eConsults is modest or poor due to study design. Patients were comfortable with eConsults, and this was increased with low-acuity conditions. PCPs are overall satisfied with eConsults. Stakeholder enthusiasm is high. Specialists are ambivalent due to inadequate compensation, liability concerns, and repeated questions by PCPs. There is a high publication bias in favour of positive studies. | Consultation, consultation and referral, remote consultation, systematic review, telemedicine |
| Whittington et al. | 2021 USA | The American Journal of Managed Care | Health services | Retrospective audit | Multispecialty | N/A | Assess the differences in cost between eConsults and face-to-face visits | Urology eConsults are associated with lower costs than face-to-face visits. However, the total costs for face-to-face visits were the lowest of all 11 specialties examined. The majority of cost savings using eConsults are due to savings in outpatient costs. | Nil |
| Witherspoon et al. | 2017 Canada | Canadian Urological Association Journal | Surgical | Mixed-methods - retrospective audit + survey | Urology | 190 | Assess the trends in urologic eConsults and their effects on referral patterns | eConsults are associated with rapid completion and response times. eConsults altered PCP referral and management behaviour. A face-to-face visit was still required in a minority of cases. eConsults were highly acceptable to PCPs | Nil |
| Zuchowski et al. | 2015 USA | Journal of General Internal Medicine | Medicine | Cross-sectional study + qualitative | Multispecialty | N/A | Assess the difficulties in communication between PCPs + specialists in the VHA | The standard EMR face-to-face referral system is limited by communication and administration difficulties. eConsults noted to be a significant improvement on this system. | Primary care, specialty care, communication, electronic health record, veterans |
